# Identification and functional analysis of protein secreted by *Alternaria solani*

**DOI:** 10.1371/journal.pone.0281530

**Published:** 2023-03-06

**Authors:** Chen Wang, Jinhui Wang, Dai Zhang, Jianing Cheng, Jiehua Zhu, Zhihui Yang

**Affiliations:** 1 College of Plant Protection, Hebei Agricultural University, Baoding, P. R. China; 2 Technological Innovation Center for Biological Control of Crop Diseases and Insect Pests of Hebei Province, Baoding, P. R. China; University of California Riverside, UNITED STATES

## Abstract

Early blight, caused by the necrotrophic fungus *Alternaria solani*, is an important foliar disease that causes major yield losses of potato. Effector proteins secreted by pathogens to host cells can inhibit host immune response to pathogens. Currently, the function of effector proteins secreted by *A*. *solani* during infection is poorly understood. In this study, we identified and characterized a novel candidate effector protein, AsCEP50. AsCEP50 is a secreted protein that is highly expressed throughout the infection stages of *A*. *solani*. *Agrobacterium tumefaciens*-mediated transient expression in *Nicotiana benthamiana* and tomato demonstrated that AsCEP50 is located on the plasma membrane of *N*. *benthamiana* and regulates senescence-related genes, resulting in the chlorosis of *N*. *benthamiana* and tomato leaves. *Δ50* mutants were unaffected in vegetative growth, spore formation and mycelium morphology. However, the deletion of AsCEP50 significantly reduced virulence, melanin production and penetration of *A*. *solani*. These results strongly supported that AsCEP50 is an important pathogenic factor at the infection stage and contributes to the virulence of *Alternaria solani*.

## Introduction

*Alternaria solani* is a necrotrophic fungus that can cause early blight disease of tomato, potato, tobacco, and many other vegetables and crops, and lead to huge losses in agricultural production [1–3]. The typical symptoms are dark brown to black lesions with concentric rings on the leaves, which can cause browning and abscission in severe cases. During the infection, *A*. *solani* digests host cell inclusions by producing germ tubes and secreting various metabolites, thereby penetrating host tissues or directly invading stomata or wounds adjacent to epidermal cells [4, 5]. In the host cell, the metabolites of *A*. *solani* also reduces the photosynthetic rate in infected leaves by inhibiting the photosystem II activity and reducing the chlorophyll and other photosynthetic pigment contents, resulting in a general reduction in plant growth and a decline in yield [6, 7].

Necrotrophic pathogens extract nutrients from dead hosts by destroying host tissues cells. Thus, cell death also occurs early in necrotrophs infection and is an indicator of successful infection [8–10]. Necrotrophic pathogens may thrive by subverting the resistance mechanisms acquired by plants to combat other pathogens [11, 12]. Necrotrophic pathogens are classified into host-specific and broad host-range species based on the number of hosts. Host-specific necrotrophs (HSNs) produces host-specific toxins (HSTs), which are critical for their pathogenicity and virulence [13, 14]. The infection mechanisms deployed by broad host-range necrotrophs (BHNs) are complex. BHNs establish compatible interactions with hosts by deploying multigene infection strategies, rendering simply inherited resistance ineffective to fight off this pathogen [15]. The fungal pathogens *Botrytis cinerea*, *Alternaria solani*, *Plectosphaerella cucumerina*, and *Sclerotinia sclerotiorum* all belong to BHNs [16–18]. In contrast to biotrophic pathogens, BHNs lacks a widely accepted working model of host-plant interactions.

Many necrotrophic pathogens utilize plant defense responses to enhance pathogenicity and induce host cell death by secreting toxic secondary metabolites [19, 20]. Some low molecular weight metabolites and secreted proteins are the basic determinants of pathogenicity or virulence [21]. Effectors enable pathogens to successful overcome microbe-associated molecular patterns (MAMPs)-triggered immunity, resulting in effector-triggered susceptibility. Likewise, these effectors can be recognized by intracellular receptors (R proteins), which activate effector-triggered immunity (ETI) [22–24]. As a key immune mechanism, ETI is associated with multiple signaling pathways in plants, and its effectors are more likely to reflect pathogenic processes in the host [25]. The effectors are utilized by necrotrophs to promote disease [26]. These results indicate that the infection mechanism of necrotrophic pathogens differs from the general theory. Therefore, the identification of *A*. *solani* effectors is of great significance to further explore the pathogenic mechanism of necrotrophic pathogens.

Based on the transcriptome and genome results of *Alternaria solani*, we selected a novel candidate effector protein, AsCEP50, with high expression levels. The AsCEP50 was localized to the host plasma membrane and its expression level increased continuously during infection. Transient expression assays in *Nicotiana benthamiana* and tomato showed that AsCEP50 regulated host senescence-related genes, resulting in chlorosis. In addition, we compared the phenotypic and pathogenic differences between wild-type, *Δ50* mutant and revertant strains. These results elucidate the role of *AsCEP50* in the vegetative growth and host infection stages of *A*. *solani*, and provide insight into the molecular interactions between *A*. *solani* and its host.

## Materials and methods

### Plants, strains materials and culture conditions

All the plants were grown on the campus of Hebei Agricultural University (Baoding, China). *Nicotiana benthamiana* and tomato were grown in a greenhouse at 23°C to 25°C with a cycle of 16-h light/8-h darkness. Potato ‘Favorita’ was grown in a greenhouse at 19°C to 22°C. The wild-type strain HWC-168 of *A*. *solani* was collected from leaves infected with potato early blight in Weichang County, Hebei Province, China. The HWC-168 was maintained in our laboratory and grown on potato dextrose agar (PDA) medium at 25°C. *Escherichia coli* strain DH5α was cultured in Luria-Bertani (LB) medium at 37°C and used for plasmid amplification. *Agrobacterium tumefaciens* strain EHA105 was cultured in LB medium at 28°C for *Agrobacterium*-mediated transient expression in *N*. *benthamiana* leaves.

### Sequence analysis

The Online tools SignalP 5.0 Server (http://www.cbs.dtu.dk/services/SignalP/), SMART (http://smart.embl-heidelberg.de/), ProParam (https://web.expasy.org/protparam/) were used to predict protein signal peptides, structural domains, molecular masses and theoretical isoelectric points.

### Vector construction

A full-length open reading frame (referred to as FL) and a truncated coding region without the signal peptide sequence but with an engineered ATG start codon (referred to as NSP) of *AsCEP50* were PCR amplified from the cDNA of potato leaves infected with HWC-168 using an *Easy Pure* Plant RNA Kit (TransGen Biotech) and a *TransScript* One-Step gDNA Removal and cDNA Synthesis SuperMix (TransGen Biotech). The hygromycin B resistance gene (*Hyg*) was amplified from the pUC-Hyg vector. The upstream and downstream sequences of *AsCEP50* gene were amplified from HWC-168 genomic DNA. The pCAMBIA1301::EGFP vector was digested with restriction enzymes SalⅠ and BamHⅠ. Recombinant Agrobacterium was constructed by ligating *AsCEP50* (FL and NSP) to the linearized pCAMBIA1301::EGFP vector using a pEASY-Uni Seamless Cloning and Assembly Kit (TransGen Biotech) for *Agrobacterium*-mediated transient expression in *N*. *benthamiana*. The *Hyg* gene, the upstream and downstream fragments of *AsCEP50* gene were connected with the pUC19 cloning vector with restriction enzymes BamHⅠ and EcoRⅠ for the construction of deletion strains. The *AsCEP50* gene was ligated with the KN vector containing the neomycin resistance gene (*Neo*) with restriction enzymes BamHⅠ and EcoRⅠ for the construction of revertant strains.

### Quantitative real-time PCR analysis

According to the research on *A*. *solani*, we selected a relatively stable housekeeping gene "*Actin*" as the internal reference gene [27, 28]. Specific primers for *Actin* and *AsCEP50* were designed using the National Center for Biotechnology Information (NCBI) database (https://www.ncbi.nlm.nih.gov/). PP2A was selected as a housekeeping gene in *N*. *benthamiana* [29]. The SEN4, SAG12 and DHAR1 sequences were obtained from Uniport (https://www.uniprot.org/), and their specific primers were designed using Primer 3 Plus (https://www.primer3plus.com/). The cDNAs of the hyphae and growing on potato and *N*. *benthamiana* leaves were serial diluted to detect primer specificity. The RNAs were extracted using an *Easy Pure* Plant RNA Kit and cDNA were synthesized using a *TransScript* One-Step gDNA Removal and cDNA Synthesis SuperMix. The cDNAs were obtained using an *Easy Pure* Plant RNA Kit and a *TransScript* One-Step gDNA Removal and cDNA Synthesis SuperMix. All the primers used in qPCR were tested for amplification efficiency by serial dilution. The qRT-PCR was performed using the Bio-Rad CFX384 Touch Real-Time System (Bio-Rad), and the total reaction volumn was 20 μL containing 2 μL gene specific primers, 6 μL of cDNA and 10 μL of 2×Magic SYBR Mixture (CWBIO, Jiangsu, China). The qRT-PCR was performed under the following conditions: 95°C for 30 s, followed by 40 cycles at 95°C for 5 s and 59°C for 30 s. The threshold cycle (CT) values were used to determine the fold change in transcript accumulation with the 2^−ΔΔCt^ method [30]. Six biological and three technical replicates were performed for each sample in all the qRT-PCR reactions.

### *Agrobacterium*-mediated transient expression in *N*. *benthamiana*

Recombinant Agrobacterium was cultured at 28°C, washed once with 10 mmol L^-1^ MgCl_2_, and then resuspended with an infiltration buffer containing 10 mmol L^-1^ MgCl_2_, 10 mmol L^-1^ 2-(N-morpholino) ethanesulfonic acid (pH 5.6) and 200 μmol L^-1^ acetosyringone. The optical density (OD)_600_ value of the *A*. *tumefaciens* suspension was adjusted to 0.6–0.8, and infiltrated into the leaves of *N*. *benthamiana* [31]. Empty vector (EV) was used as the negative control and INF1 was used as the positive control. Each treatment was replicated six times.

To detect HR, the strain expressing AsCEP50 protein was infiltrated into the right side of each leaf and EV was infiltrated into the left side. The *N*. *benthamiana* and tomato were cultured at 23°C for 5 days until symptoms appeared. Whole *N*. *benthamiana* and tomato leaves were immersed in 3,3’-diaminobenzidine (DAB) solution (CWBIO, Jiangsu, China) at room temperature. After 12 hours, the leaves were soaked in 95% ethanol and boiled for 30 min and photographed. Each treatment was repeated six times.

For subcellular localization, EV and *AsCEP50*-NSP expression vectors were separately infiltrated into 5-week-old *N*. *benthamiana* leaves. At 48-72 hours post inoculation (hpi), the leaves epidermal cells were visualized with excitation at 488 nm and emission at 507 nm using a confocal laser scanning microscope (FV10I, Olympus, America).

### Signal peptide secretion assay

The function of the predicted signal peptides was verified by the yeast secretion system. To determine the signal peptide secretory activity, the signal peptide of AsCEP50 was cloned into vector pSUC2 using specific primers. The recombinant vector was transformed into yeast strain YTK12. The positive colonies were screened on a CMD−W medium (0.075% tryptophan dropout supplement, 0.67% yeast nitrogen base without amino acids, 2% sucrose, 0.1% glucose, and 2% agar). To test for invertase secretion, successfully transformed yeast strains were grown on YPRA agar (1% yeast extract, 2% peptone, 2% raffinose, 2 mg/mL antimycin A, and 2% agar).

### Deletion and complementation of *AsCEP50*

The *AsCEP50* gene was replaced with *Hyg* using homologous recombination technology. Approximately 1-kb upstream and downstream fragments from the wild-type HWC-168 genomic DNA were amplified and linked to *Hyg* gene to construct the *Δ50* deletion mutant strains. The fusion fragments were used for the protoplast mediated transformation of HWC-168.

The wild-type HWC-168 hyphae were inoculated into 200-mL flasks containing 100 mL Potato Dextrose Broth (PDB) medium, incubated at 25°C and shaken at 120 rpm for 36 h. The mycelia were filtered through three layers of gauze and thoroughly rinsed with 0.7 mol L^-1^ NaCl solution 2–3 times. The hyphae were transferred to a 50-mL centrifuge tube using tweezers, and digested with a mixture of enzymes, 0.1 mg of snailase (Solarbio, Beijing, China), 0.1 mg of driselase (Biotopped, Beijing, China) and 0.1 mg of lysing enzyme (SIGMA, Shanghai, China) in 10 mL of 0.7 mol L^-1^ NaCl. The protoplasts were resuspended in STC buffer (1.168 M D-Sorbitol, 10 mM Tris-HCl and 4.96 mM CaCl_2_) and the fusion fragments mixtures were added to the PTC buffer (15 mM PEG4000, 10 mM Tris-HCl and 10 mM CaCl_2_). The transformants were screened on Regeneration PDA medium containing ampicillin (50 μg mL^–1^) and hygromycin (50 μg mL^–1^), and PCR was performed using specific primers of *Hyg*, upstream and downstream fragments to confirm the transformants.

After obtaining the mutant strains, the protoplasts were obtained from the *Δ50* mutant strains. The recombinant KN vector was transformed into the protoplasts of mutant strains by PEG-mediated protoplast transformation. The neomycin resistance was used to screen the revertant strains.

### Detection of melanin, penetration and pathogenicity

The colonies of the wild-type, *Δ50* mutant and revertant strains with a diameter of 5 mm were inoculated independently into the 350-mL flasks containing 100 mL PDB medium, incubated at 25°C and shaken at 150 rpm for 5 d. The solutions of the wild-type, *Δ50* mutant and revertant strains were filtered with gauze, and the absorbance of filtrate was measured at 400 nm.

Sterile cellophane was spread on the PDA medium, and then, wild-type, deletion mutant and revertant strains were placed on the PDA medium at 25°C for 3 d in the dark. Then, the cellophane was uncovered and the strains were cultured for 4-5 d.

The HWC-168 strain was inoculated into tomato agar medium. After the hyphae were overgrown, mechanical damage was performed, and sporulation was stimulated by ultraviolet light for 15 min. Spore suspensions of HWC-168 wild-type, *Δ50* mutant and revertant strains were prepared and their concentrations were adjusted to 10^5^ mL^-1^. Five-week-old potato leaves were sterilized with 75% alcohol, rinsed with sterile water, and then the petioles were inserted into water agar medium. Approximately 20 μL of each suspension was inoculated onto potato leaves *in vitro*. The leaves were incubated at 25°C under a 16-h photoperiod. The accumulation of H_2_O_2_ was detected by DAB staining [32]. The symptomatic potato leaves were soaked in DAB solution and left in the dark for 12 h. Three independent experiments were performed, each with six biological repeats.

## Result

### Bioinformatics identification of AsCEP50

We performed functional analysis of the *AsCEP50* gene (GenBank accession numbers: OM735615) with effector characteristics. The signal peptide of *AsCEP50* gene correponded to 1-19 amino acids (aa) using SignalP analysis (S1 Fig). SMART protein analysis revealed that AsCEP50 was composed of Pro_Al_protease domain (amino acid positions 107 to 174) and Trypsin domain (amino acid positions 197 to 369) (S2 Fig). ProtParam was used to analyze the physicochemical properties of AsCEP50 protein (S1 Table).

### High expression of *AsCEP50* in the infection

**Fig 1:**
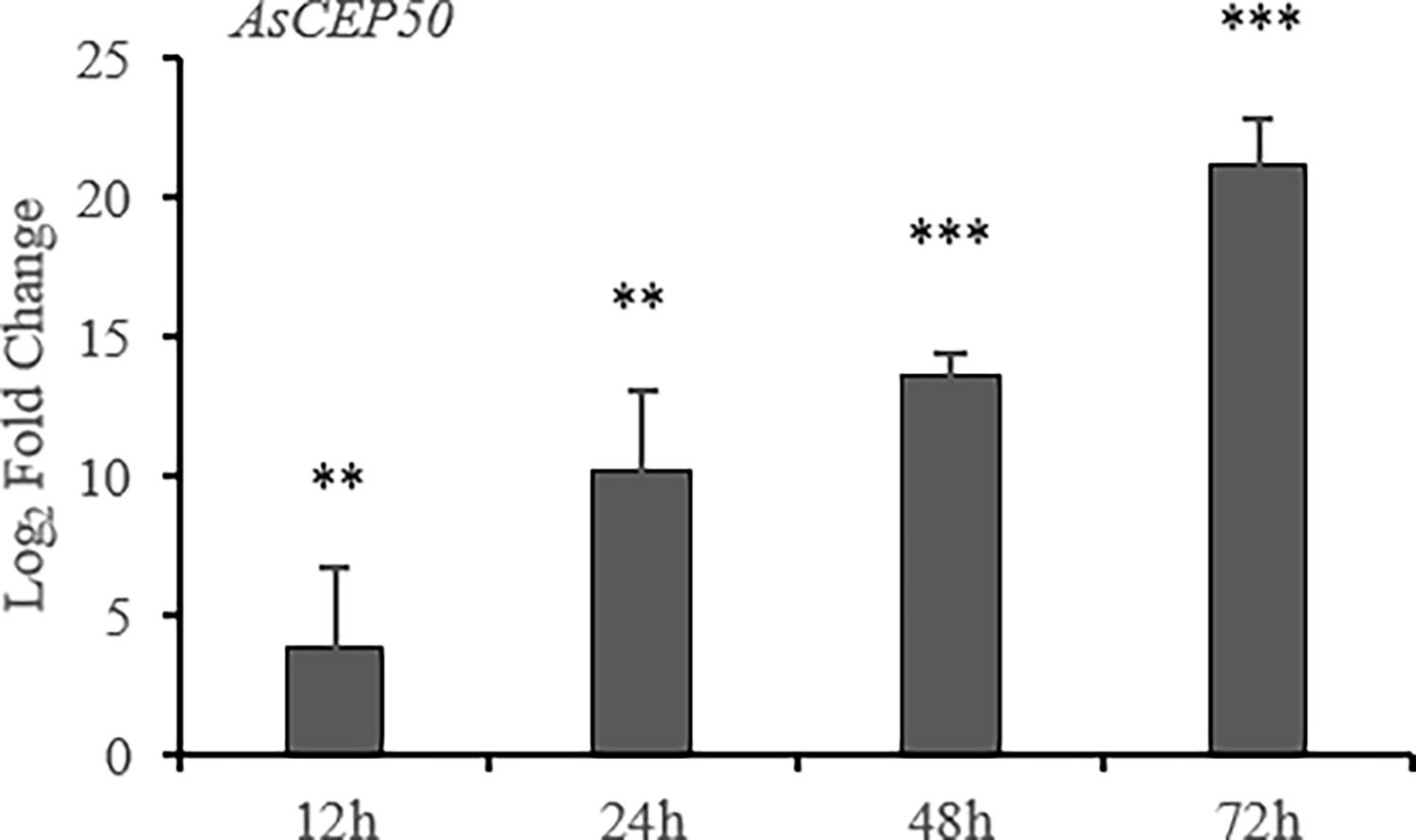
Expression patterns of the *AsCEP50* gene during the infection of potato leaves. The expression level at the hyphal stage was used as a control to analyze the specific expression at the infection stage. Student’s t-test was used as a significance test of difference (n=6). **, indicates a significant difference (0.001<p<0.01) compared with the control (hyphae stage); ***, indicates an extremely significant difference (p<0.001) compared with the control. Error bars represent the standard deviation (SD) of four biological replicates and two technical replicates.

AsCEP50 is a single-copy gene in the *A*. *solani* genome. To determine the function of *AsCEP50* in the infection of *A*. *solani*, the temporal expression pattern of AsCEP50 in infected potato leaves was revealed by qRT-PCR using *A*. *solani* mycelium as a control (Fig 1). The expression of *AsCEP50* in the infection stage continued to increase compared with the hyphal stage, and the log_2_-fold change of *AsCEP50* expression was increased by 21.16-fold, with all p-values less than 0.01.

### AsCEP50 has a secretory function and is localized to the *N*. *benthamiana* plasma membrane

The predicted secretory function of the signal peptide was confirmed by a yeast invertase secretion assay [33]. The N-terminal 19 amino acids of AsCEP50 are predicted by SignalP 5.0 to be a specific signal peptide sequence. When the predicted signal peptide of AsCEP50 was fused to the yeast invertase sequence in the vector pSUC2, it mediated the complementation of yeast YTK12 mutants (invertase-deficient) grown on raffinose or YPRA agar. The results show that the predicted signal peptide of AsCEP50 is functional (S3 Fig).

**Fig 2:**
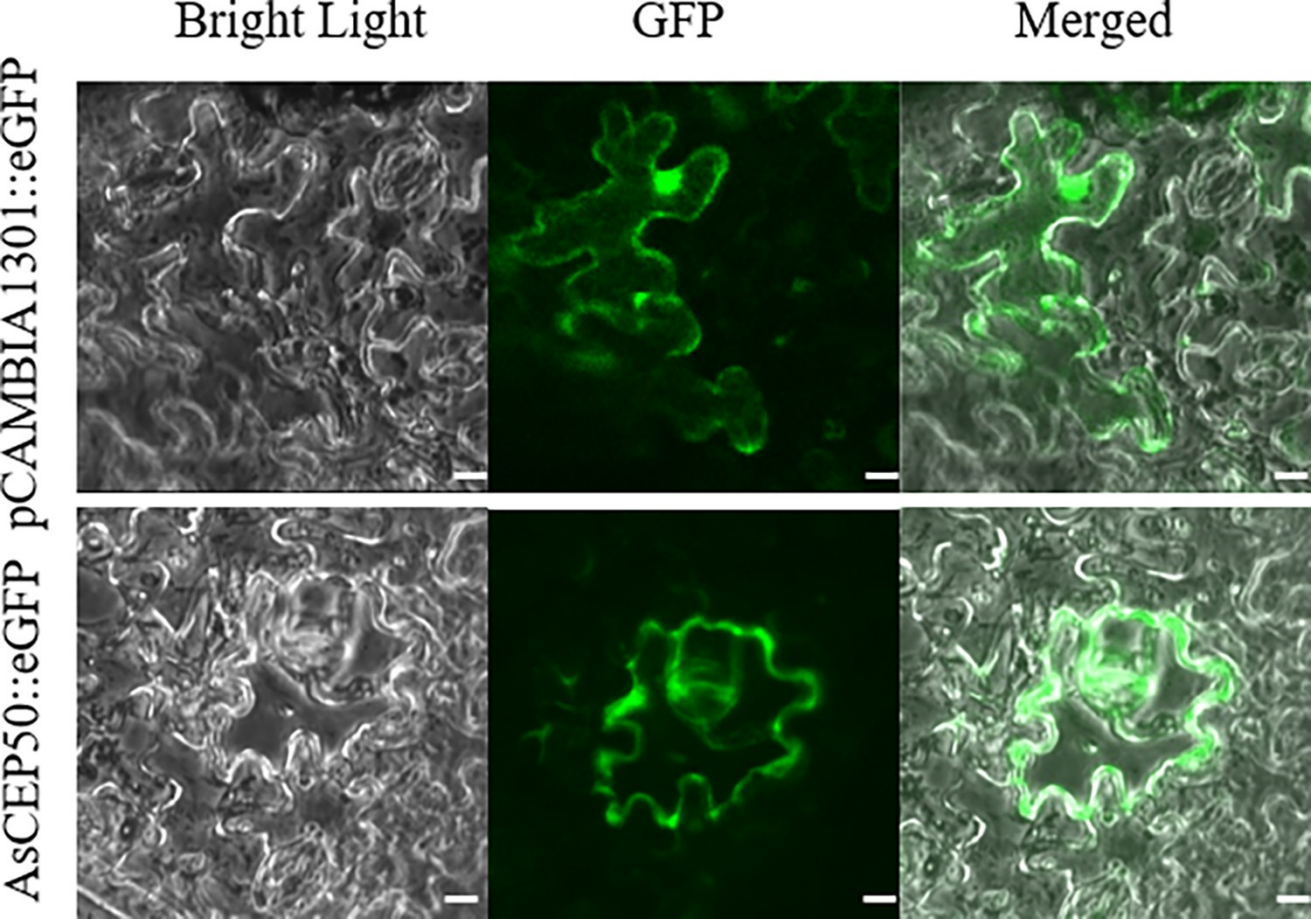
Subcellular localization of *AsCEP5*0 proteins in *N*. *benthamiana*. The *Agrobacterium* strain EHA105 containing the pCAMBIA1301 vector as the control and the *AsCEP50* gene were transiently expressed in the leaves of *N*. *benthamiana*. Bar=20 μm.

Effectors are secreted into host cells, where they localize to specific subcellular compartments to perform different activities [34, 35]. Secreted effectors can target different organelles and interfere with host immunity by inhibiting the translation of receptor proteins [36]. We attempted to determine the subcellular localization of AsCEP50 when expressed in plant cells. Therefore, to detect the target of AsCEP50 in *N*. *benthamiana* cells, *AsCEP50*-NSP was cloned into pCAMBIA1301::EGFP. As shown in Fig 2, the fluorescence signal of AsCEP50-NSP appeared on the plasma membrane. These results indicated that AsCEP50 played a role in the plasma membrane of *N*. *benthamiana* cells.

### AsCEP50 promotes chlorosis of *N*. *benthamiana* and tomato leaves

Fungal pathogens manipulate the host immune response to facilitate infection by secreting specific effector proteins. Transient expression analysis was performed to determine whether AsCEP50 directly induced host cell death, suppressed host immune responses, or promoted host cell death in *N*. *benthamiana*. INF1 induces programmed cell death (PCD) in *N*. *benthamiana* as a positive control [37]. When AsCEP50 (FL and NSP) was injected alone or co-injected with INF1, there was no symptoms of directly inducing host cell death and suppressing the host immune responses (S4 Fig).

**Fig 3:**
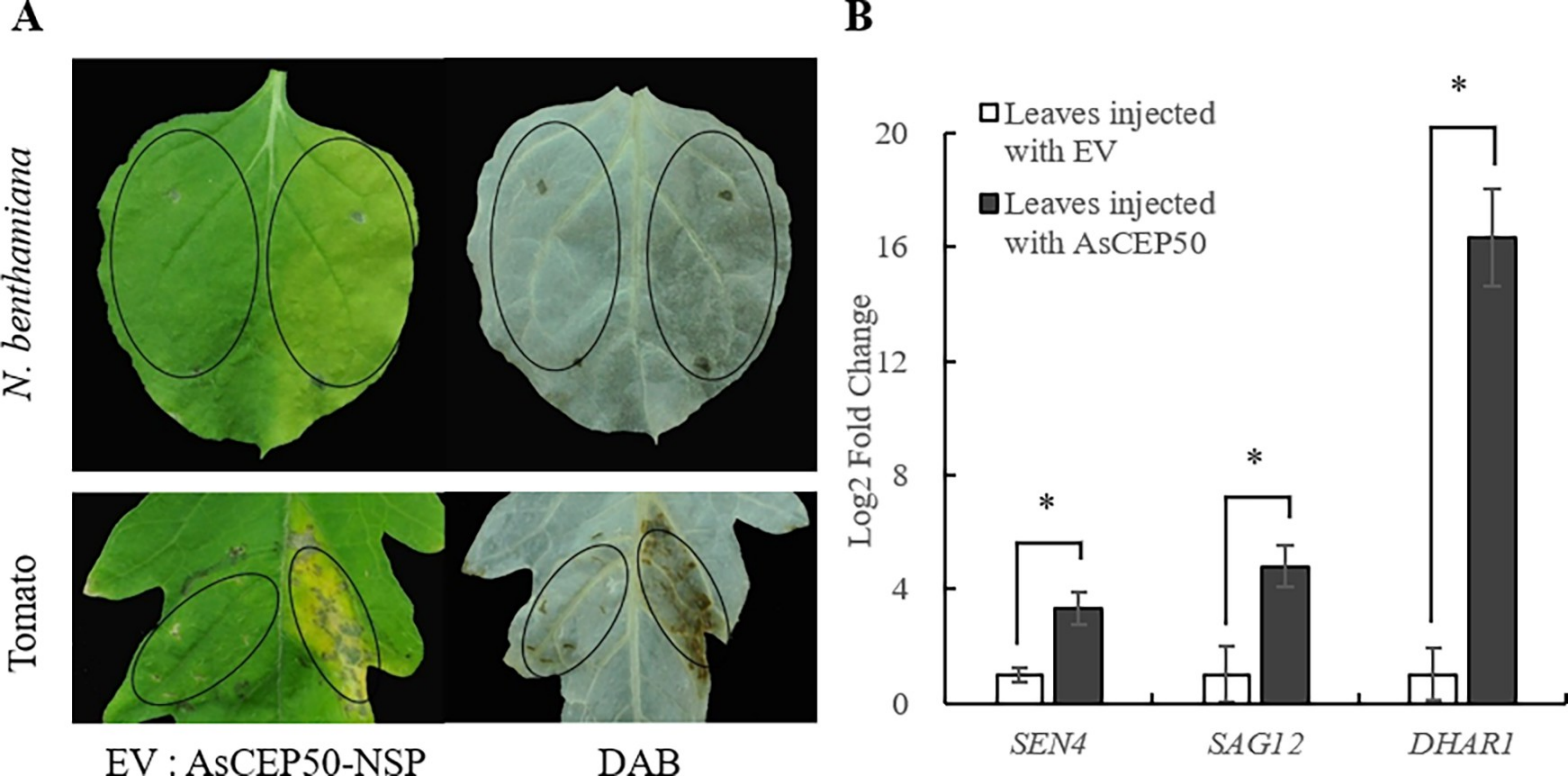
*Agrobacterium* containing AsCEP50 effector promotes senescence of *N*. *bethamiana* and tomato leaves. (**A**) *Nicotiana benthamiana* and tomato leaves were infiltrated by control (EV; left, leaf tip to petiole direction) and pCAMBIA1301 *Agrobacterium* containing *AsCEP50*-NSP gene (right, leaf tip to petiole direction). (**B**) Relative mRNA levels of *SEN4*, *SAG12* and *DHAR1*. Expression levels of senescence- and oxidative stress-associated genes were examined in *N*. *benthamiana* (EV and *AsCEP50*-NSP) 4 days after injection with *Agrobacterium*.

In addition, we further verified whether AsCEP50 has the function of promoting infection. The pCAMBIA1301 vector was used as a negative control. Chlorotic symptoms were observed in the leaves infiltrated with *Agrobacterium* AsCEP50-NSP and AsCEP50-FL alone. At the same time, we repeated this experiment on tomato leaves and found that *Agrobacterium* AsCEP50-NSP and AsCEP50-FL also caused tomato leaf chlorosis. Furthermore, we investigated the ability of AsCEP50-NSP and AsCEP50-FL to stimulate hydrogen peroxide (H_2_O_2_) in *N*. *benthamiana* and tomato leaves. The results showed that AsCEP50-NSP and AsCEP50-FL accumulated more H_2_O_2_ than controls at 5 dpi (Fig 3).

Many genes that regulate senescence during plant growth have been defined as senescence related genes. The expression levels of three senescence-related genes (*SEN4*, *SAG12*, and *DHAR1*) were determined in EV and AsCEP50-infiltrated leaves. After treatment with AsCEP50, the expression levels of senescence-related genes were significantly up-regulated compared with EV treatment. The expression level of *DHAR1* was 16 times higher than EV treatment. These results suggested that AsCEP50 induced plant leaf chlorosis by regulating senescence-related genes.

### Generation and validation of *AsCEP50* deletion and revertant strains

**Fig 4:**
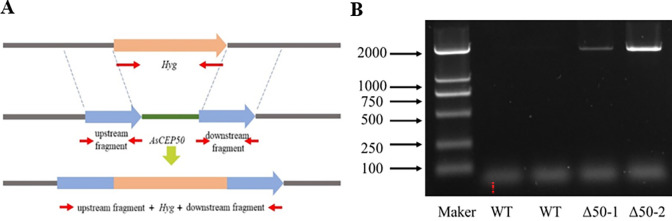
*AsCEP50* gene replacement and PCR screening of mutants. (**A**) The diagram of homologous recombination of *AsCEP50* and *Hyg* gene. (**B**) Verification of *Hyg* gene in the genomes of wild-type and *Δ50* mutant strains.

The *AsCEP50* gene was replaced by *Hyg* gene using PEG-mediated protoplast transformation. Two hygromycin-resistant *A*. *solani* strains were obtained by three independent transformation processes on selective plates containing ampicillin (100 μg/mL) and hygromycin (100 μg/mL). The *Hyg* gene was successfully amplified from two AsCEP50 deletion strains by RT-PCR using *Hyg-*specific primers, but no *Hyg* gene was amplified in wild-type strain (Fig 4). Thus, *AsCEP50* was replaced by a single copy of *Hyg* in the genomic DNA of *A*. *solani*.

In the same manner, the *Hyg* gene in each *Δ50* mutant strain was replaced by *AsCEP50* gene, and two revertant strains corresponding to *Δ50* mutant strains were obtained (S5 Fig).

### *AsCEP50* affects colony melanin and penetration, but not the growth and sporulation rates

To test the role of *AsCEP50* in the growth of *A*. *solani*, we compared the colony morphology, growth rate, sporulation rate and penetration of wild-type, *AsCEP50* deletion and revertant strains. The colony growth and spore formation rates of wild-type, two *AsCEP50* deletion and two revertant strains were similar (S6 Fig).

**Fig 5:**
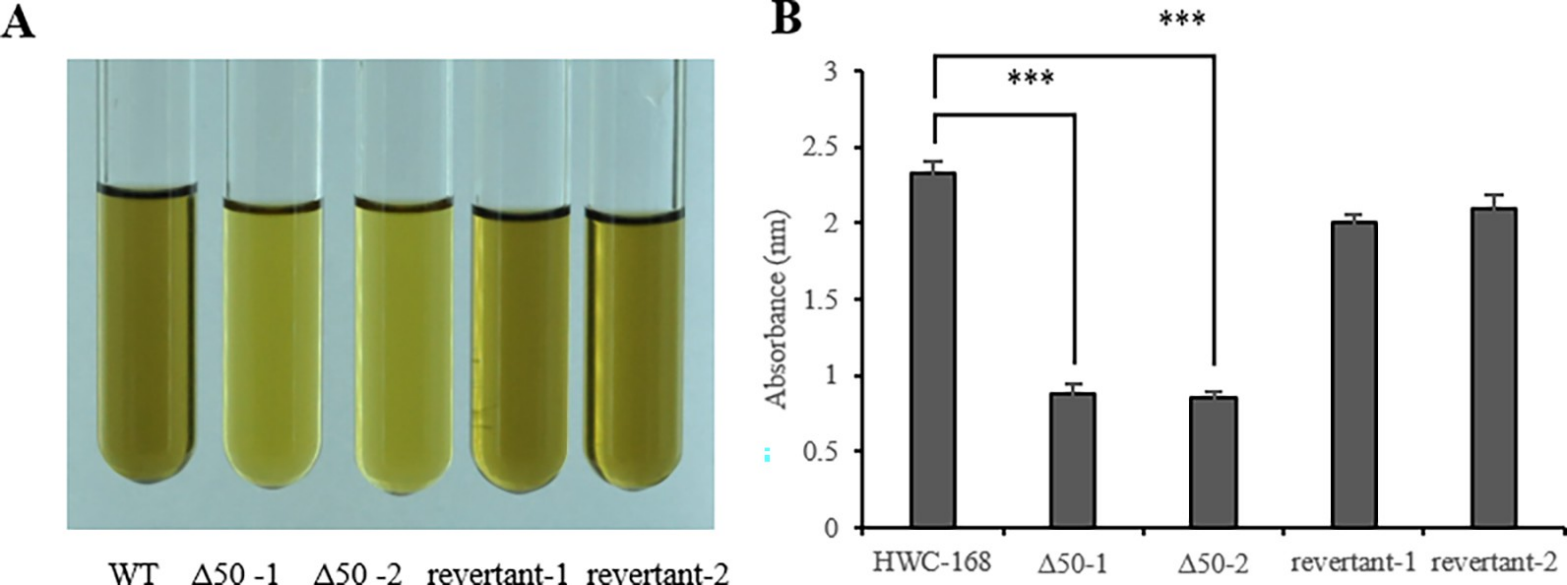
Determination of the phenotype and melanin of the wild-type, *Δ50* mutant and revertant strains. (**A**) the solution of wild-type, *Δ50* mutant and revertant strains that determine the melanin content. (**B**) the absorbance of the wild-type, *Δ50* mutant and revertant strain solutions at 400 nm.

The melanin area in the inner circles of the *Δ50* mutant strain colonies were smaller compared with those of the wild-type and revertant strains (S7 Fig). Therefore, the melanin content of the wild-type, *Δ50* mutant and revertant strains were detected. The filtrate of *Δ50* mutant strains became lighter in color. Then, the absorbance value of *Δ50* mutant strains was significantly decreased by filtrate OD_400_ (Fig 5). The results indicated that *AsCEP50* deficiency reduced the melanin content of *A*. *solani*.

**Fig 6:**
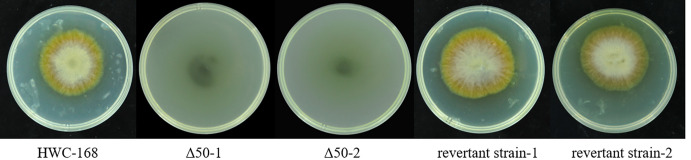
Penetration of the WT, *Δ50* mutant and revertant strains. The colony morphology of WT (left), *Δ50* mutant (middle) and revertant strains (right) filtered through three layers of sterile cellophane.

Mechanical penetrations of wild-type, *AsCEP50* deletion and revertant strains were also compared. When three layers of cellophane were spread on PDA medium, the cellophane was penetrated by the wild-type and revertant strains. The *Δ50* mutant strains left melanin on PDA medium, but they did not penetrate the cellophane (Fig 6). These results suggested that the deletion of *AsCEP50* also reduced the penetration of *A*. *solani*.

### Deletion of the *AsCEP50* results in a significant reduction in virulence

**Fig 7:**
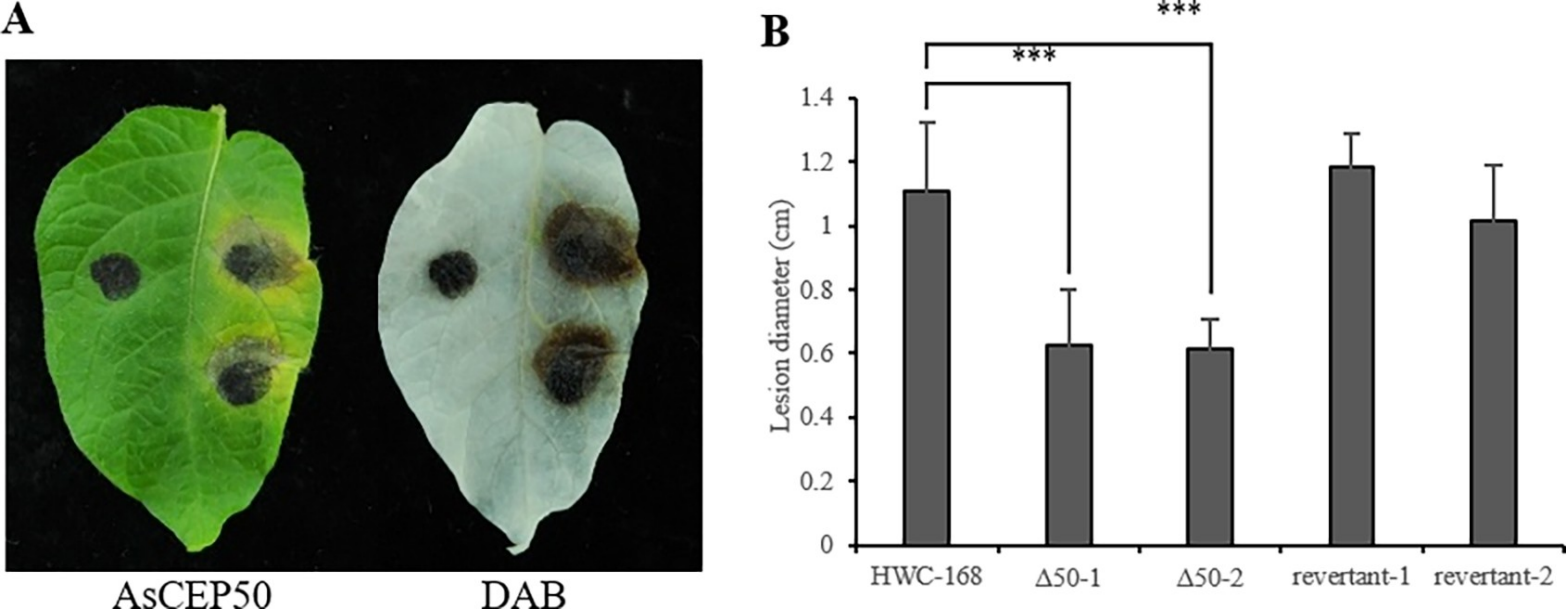
Pathogenicity detection of *AsCEP50* gene. (**A**) The detached potato leaves were inoculated with the spore suspensions of *Δ50* mutant strains (left, leaf tip to petiole direction), wild-type strains (upper right, leaf tip to petiole direction) and revertant strains (lower right, leaf tip to petiole direction). (**B**) The lesion diameters of potato leaves infected by the wild-type, *Δ50*-1 mutant, *Δ50*-2 mutant, revertant-1 and revertant-2 strains. ***, indicates an extremely significant difference (p<0.001) compared with the control (HWC-168). Bar means standard deviation (SD).

To determine whether the deletion of *AsCEP50* affected the full virulence of *A*. *solani*, the detached leaves of potatoes were treated with spore suspensions of wild-type, *Δ50* mutant and revertant strains. The results showed that the average diameter of the lesions of the *Δ50* mutant strain was approximately 56% that of wild-type strain. The average diameter of lesions of the WT strain was approximately 1.11 cm. The average diameters of lesions of the mutant strains were approximately 0.63 and 0.62 cm, and the average diameters of lesions of the revertant strains were approximately 1.18 and 1.01 cm. The *AsCEP50* deletion strains had a smaller lesion area and less chlorosis in leaves compared with the wild-type and revertant strains (Fig 7). In addition, the infected leaves were soaked in DAB solution for staining, which showed that H_2_O_2_ accumulations of the *Δ50* mutant strains at the inoculation sites were lower than that of wild-type and revertant strains (Fig 7A right panel). The deletion of *AsCEP50* reduced the virulence of *A*. *solani* in infection of potato leaves. The experiment was completed six independent times with similar results. Each experiment was 6-10 biological replicates (S8 Fig).

## Discussion

*Alternaria solani* is a kind of necrotrophic pathogen that can cause early blight disease of tomato, potato, tobacco, and many other vegetables and crops, and lead to huge losses in agricultural production [1]. The changes in the JA- and SA-dependent defenses pathways in the host during an *A*. *solani* infection are different from those that occur during common necrotroph infections [38]. To date, there have been no in-depth studies on the factors released by *A*. *solani* during potato infection. Therefore, it is very crucial to study the pathogenic mechanism of *A*. *solani* infection in potato.

Although not all secreted proteins were associated with pathogenicity, studies have shown that secreted proteins expressed during the infection phase are more likely to affect pathogenicity of pathogens [39]. Specific effector proteins can be expressed at different pathogenic stages, indicating that different effector proteins play unique roles in different stages of infection [40]. The expression of *AsCEP50* gene increased continuously within 72 h of the infection stage. The damage to host cells was most severe within 72 h after infection with *A*. *solani*. *Alternaria solani* invades host cells through hyphae by 8 hpi, and then destroys the host cells by 24 hpi. At 72 hpi, the host cells are basically eliminated. The changes in host cells during the entire infection process corresponded to the changes in *AsCEP50* expression. Additionally, the expression levels of defense-related genes and defense-related enzymes (SOD, POD and PAL) in the host also increase significantly in 72 h, indicating that the substances secreted by *A*. *solani* cause serious damage to defense-related responses of the host [41]. Therefore, the regulation of *AsCEP50* expression may indicate that *AsCEP50* plays an important pathogenic role in attacking host defense systems and digesting host cells.

Phytopathogenic effectors are often overexpressed in plants to induce phenotypes that reflect their level of virulence activity. Effectors with different functions may directly induce, inhibit or promote host cell death in the infection [42]. For example, as a typical necrotrophic pathogen, *Sclerotinia sclerotiorum* not only secretes SsCP1 effector to induce the host cell death, but also secretes SsITL effector to inhibit the host immune responses [43, 44]. The yeast signal trap assay system demonstrated that the signal peptide predicted by AsCEP50 was fused to the invertase gene, which induced yeast to secrete invertase. This indicates that AsCEP50 has a secretory function. For transient expression of AsCEP50, *N*. *benthamiana* leaves showed no direct induction of cell death and inhibition of cell death. The leaf chlorosis symptoms of *N*. *benthamiana* and tomato and the expression of senescence-related genes in the host indicated that AsCEP50 promoted leaf chlorosis by regulating the expression of senescence-related genes. There are many pattern recognition receptors (PRRs) in the host cell membrane, which play an unparalleled role in resistance to pathogen infection [45, 46]. Furthermore, a crucial defense pathway exists between plant cell membranes and chloroplasts, and disruption of this pathway ultimately disrupts SA-dependent defenses [47]. The effect of *A*. *solani* on the SA signaling pathway in host defense and the localization of the candidate effector AsCEP50 secreted by *A*. *solani* to the cell membrane of *N*. *benthamiana* suggest that AsCEP50 is likely to play an important role in infection.

Melanin is widely found in fungi, and it greatly increases the fungal tolerance to external conditions, including ultraviolet radiation, enzymatic hydrolysis and extreme temperatures [48, 49]. Although the production of melanin is not required by pathogenic fungi, it can enhance the protective function of pathogens against external stress [50–52]. The deletion of *AsCEP50* reduced the melanin content of the mutant strains and decreased hyphal penetration. Likewise, the pathogenicity of isolated potato leaves inoculated with wild-type, deletion and revertant strains showed that deletion of the *AsCEP50* gene resulted in reduced spot area and lessened virulence of *Alternaria solani*. The effector proteins such as CfEC92 in *Colletotrichum fructicola*, F12 in *Fusarium graminearum* and AvrPtoB in *Pseudomona* all play an important roles in promoting the virulence of the pathogens themselves [53–55].

In this study, we identified the *AsCEP50* gene of the *A*. *solani* candidate effector as having a critical role in infection. The *AsCEP50* gene was highly expressed throughout the infection process of *A*. *solani*, and was localized to the host cell membrane. The gene promoted host leaf chlorosis by regulating host senescence-related genes. In addition, the comparison of wild-type, mutant and revertant strains found that *AsCEP50* not only affected the melanin density and penetration of *A*. *solani*, but was also crucial for the pathogenicity of *A*. *solani*. We will further investigate the targets and interaction modes of AsCEP50 in the host to explain the molecular basis of necrotrophic pathogen-host interactions.

## Supporting information

S1 Fig Detection of AsCEP50 protein signal peptide. AsCEP50 protein 1-19 is a signal peptide sequence.
(TIF)

S2 Fig Structure of AsCEP50 protein. The AsCEP50 protein contains domains Pro_Al_protease and Trypsin.
(TIF)

S3 Fig The signal peptide (SP) of AsCEP50 is functional. The validation of the function of AsCEP50^SP^ with yeast signal trap assay. The YTK12 yeast strain containing pSUC2 is able to grow on a CMD−W medium without tryptophan, but not on YPRAA medium. AsCEP50^SP^ can grow on both CMD−W and YPRAA media. The SP of Avr1b was used as positive control.
(TIF)

S4 Fig Transient expression of *AsCEP50* in *N*. *benthamiana* leaves. The upper left and upper right corners of the leaf were injected with control (EV) and AsCEP50 (FL/NSP), respectively. The lower left and right corners were respectively injected with INF1 and AsCEP50 (FL/NSP) coupled with INF1.
(TIF)

S5 Fig *Hyg* gene replacement and PCR screening of mutant and revertant strains. Verification of the *AsCEP50* gene in the genomes of mutant and revertant strains (RT).
(TIF)

S6 Fig The colony areas and growth radii of the WT, *Δ50* mutant and revertant strains. (TIF)

S7 Fig Determination of the phenotypes of the wild-type, *Δ50* mutant and revertant strains. The colony phenotypes of *Alternaria solani* wild-type (left), *AsCEP50* mutant (middle) and revertant (right) strains cultured on PDA medium for 7 d at 25°C in the dark.
(TIF)

S8 Fig Pathogenicity detection of *AsCEP50* gene. The isolated potato leaves were inoculated with the spore suspensions of *Δ50* mutant strains (left, leaf tip to petiole direction), wild-type strains (upper right, leaf tip to petiole direction) and revertant strains (lower right, leaf tip to petiole direction).
(TIF)

S1 Table Bioinformatics-based identification of the AsCEP50 protein. (PDF)

S2 Table Expression levels of senescence- and oxidative stress-associated genes. The expression of *Actin*, *SEN4*, *SAG12* and *DHAR1* in *N*. *benthamiana* leaves injected with empty vector and *Agrobacterium* containing *AsCEP50*.
(PDF)

S3 Table Quantitative data for senescence. Lesion length and width of *N*. *benthamiana* leaves injected with empty vector and *Agrobacterium* containing *AsCEP50*.
(PDF)

S4 Table Primer amplification efficiency. (PDF)

S5 Table Primers used for this study. (PDF)

S1 Raw images (PDF)
