# Supplementary figures and images for: Identification and functional analysis of protein secreted by Alternaria solani
Source: PLoS One. 2023 Mar 6;18(3):e0281530. doi: 10.1371/journal.pone.0281530 (PMC9987770; doi:10.1371/journal.pone.0281530)

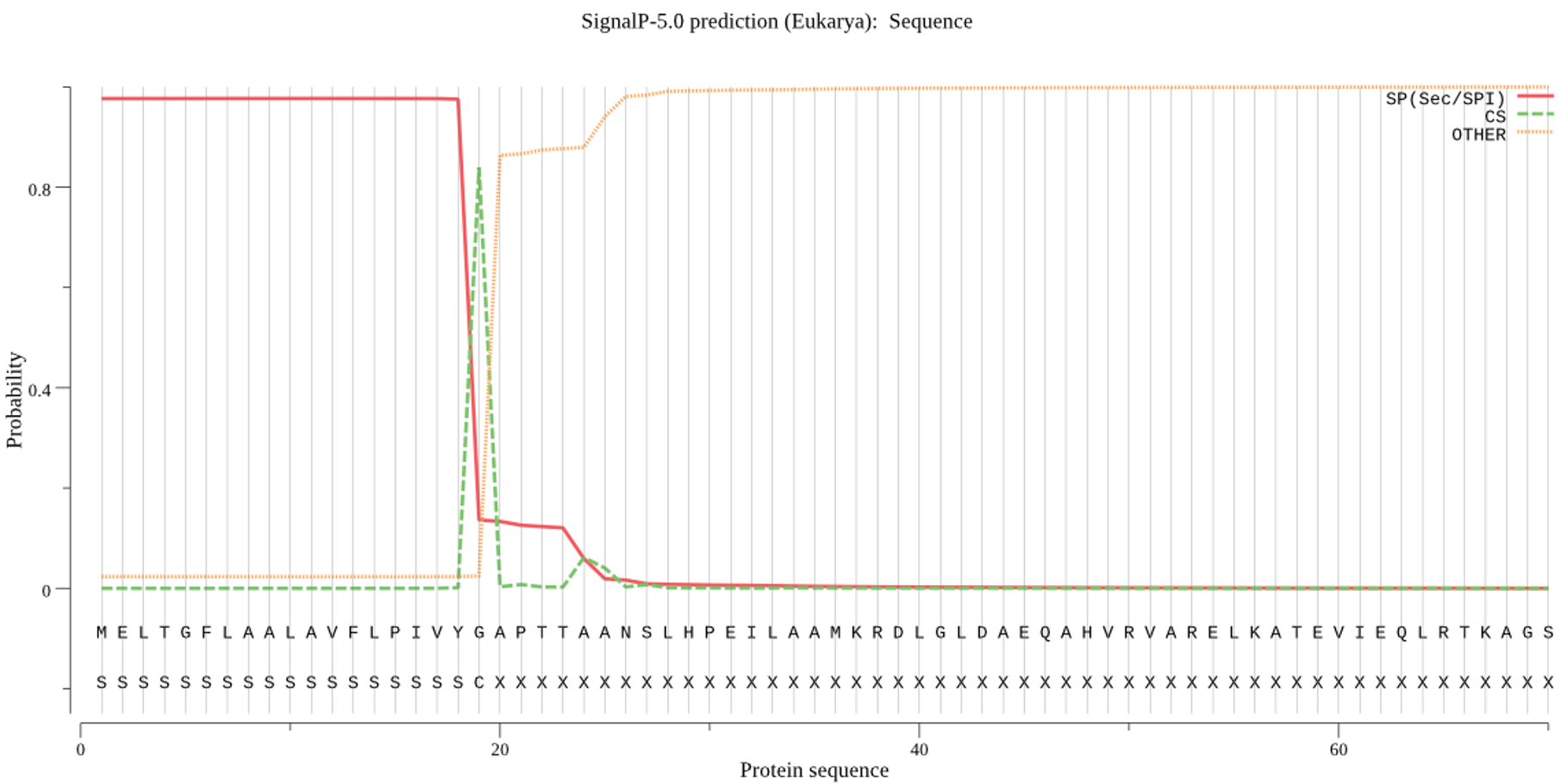

Supplement: S1 Fig — AsCEP50 protein 1-19 is a signal peptide sequence. (TIF) [file pone.0281530.s001.tif]

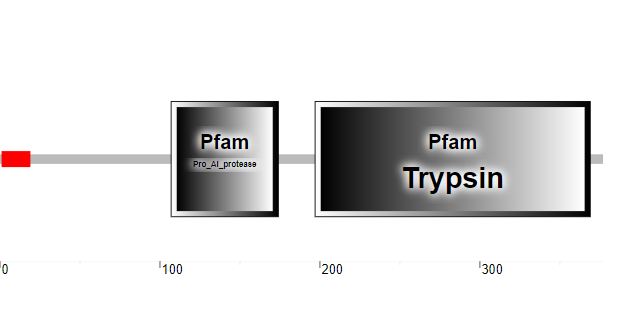

Supplement: S2 Fig — The AsCEP50 protein contains domains Pro_Al_protease and Trypsin. (TIF) [file pone.0281530.s002.tif]

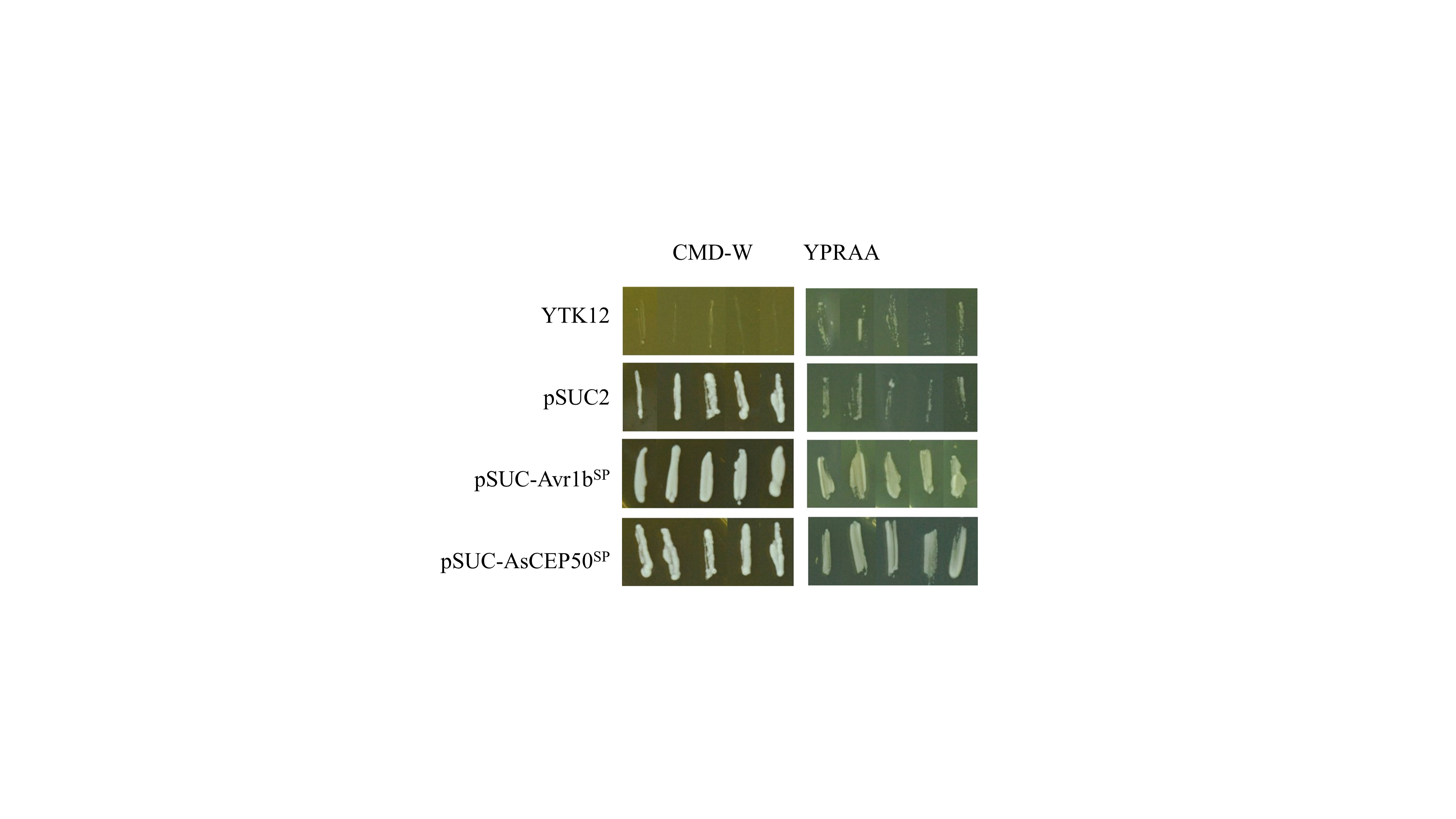

Supplement: S3 Fig — The validation of the function of AsCEP50SP with yeast signal trap assay. The YTK12 yeast strain containing pSUC2 is able to grow on a CMD−W medium without tryptophan, but not on YPRAA medium. AsCEP50SP can grow on both CMD−W and YPRAA media. The SP of Avr1b was used as positive control. (TIF) [file pone.0281530.s003.tif]

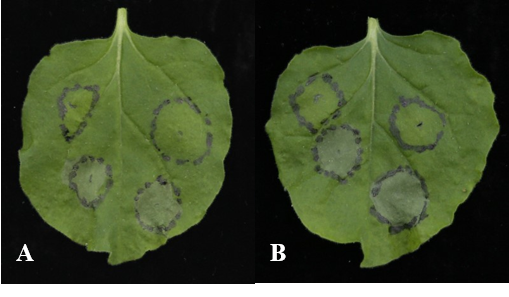

Supplement: S4 Fig — The upper left and upper right corners of the leaf were injected with control (EV) and AsCEP50 (FL/NSP), respectively. The lower left and right corners were respectively injected with INF1 and AsCEP50 (FL/NSP) coupled with INF1. (TIF) [file pone.0281530.s004.tif]

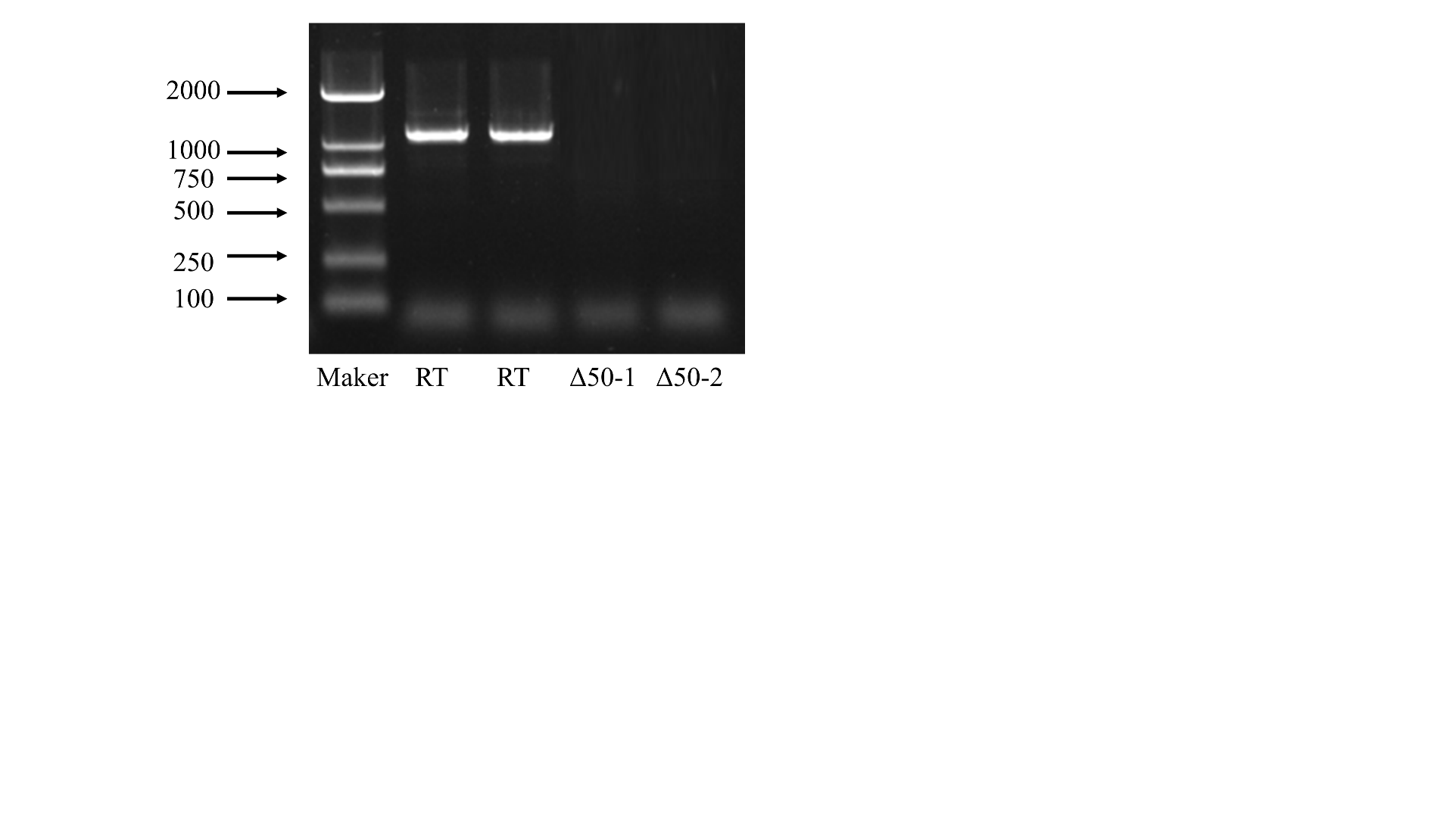

Supplement: S5 Fig — Verification of the AsCEP50 gene in the genomes of mutant and revertant strains (RT). (TIF) [file pone.0281530.s005.tif]

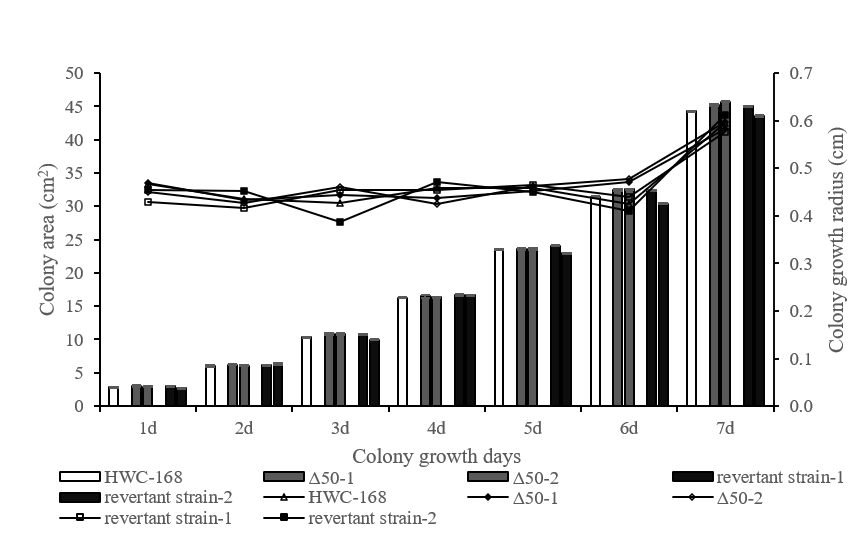

Supplement: S6 Fig — (TIF) [file pone.0281530.s006.tif]

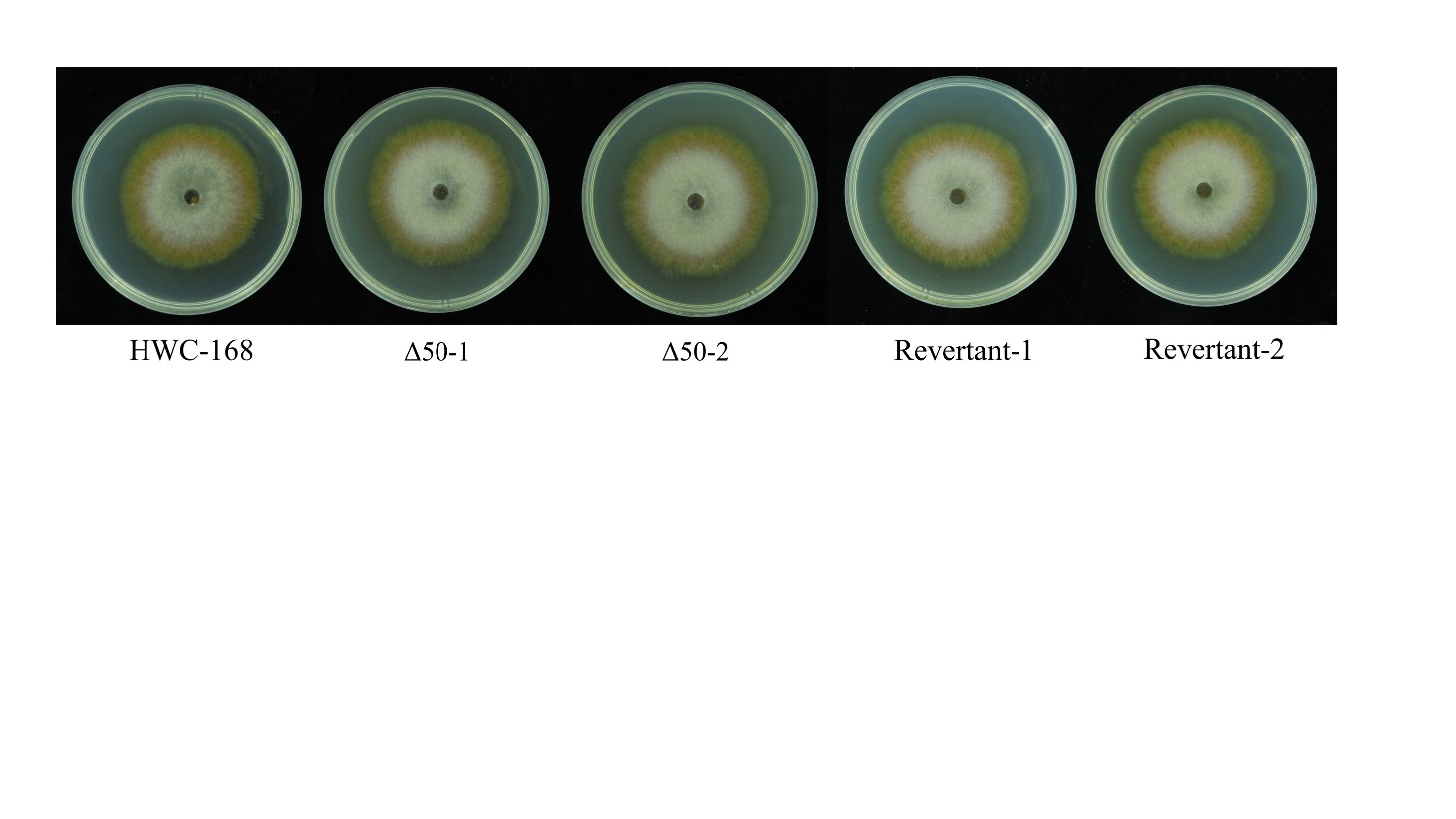

Supplement: S7 Fig — The colony phenotypes of Alternaria solani wild-type (left), AsCEP50 mutant (middle) and revertant (right) strains cultured on PDA medium for 7 d at 25°C in the dark. (TIF) [file pone.0281530.s007.tif]

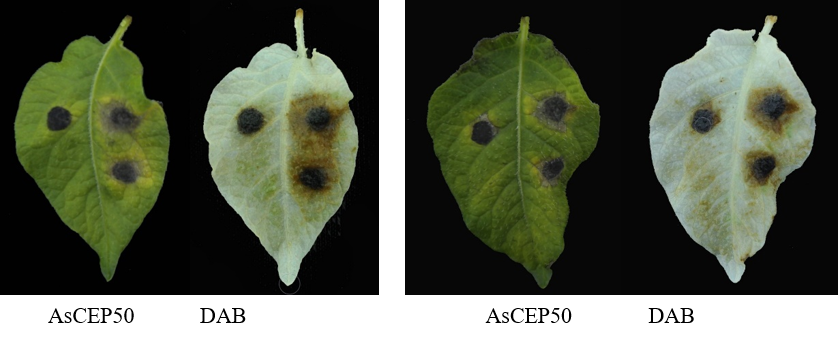

Supplement: S8 Fig — The isolated potato leaves were inoculated with the spore suspensions of Δ50 mutant strains (left, leaf tip to petiole direction), wild-type strains (upper right, leaf tip to petiole direction) and revertant strains (lower right, leaf tip to petiole direction). (TIF) [file pone.0281530.s008.tif]

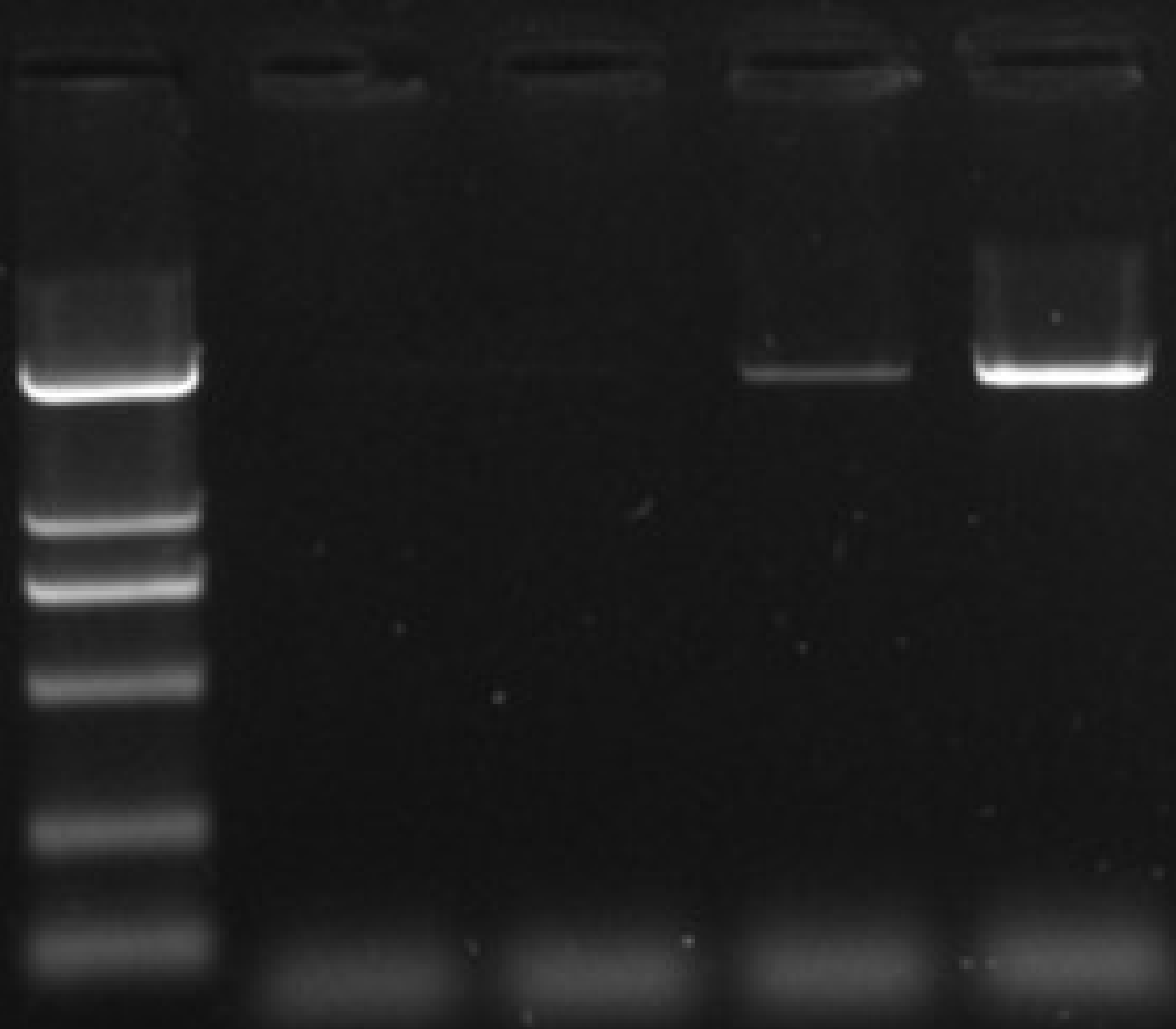

WT WT 50-1 50-2

Fig.4B

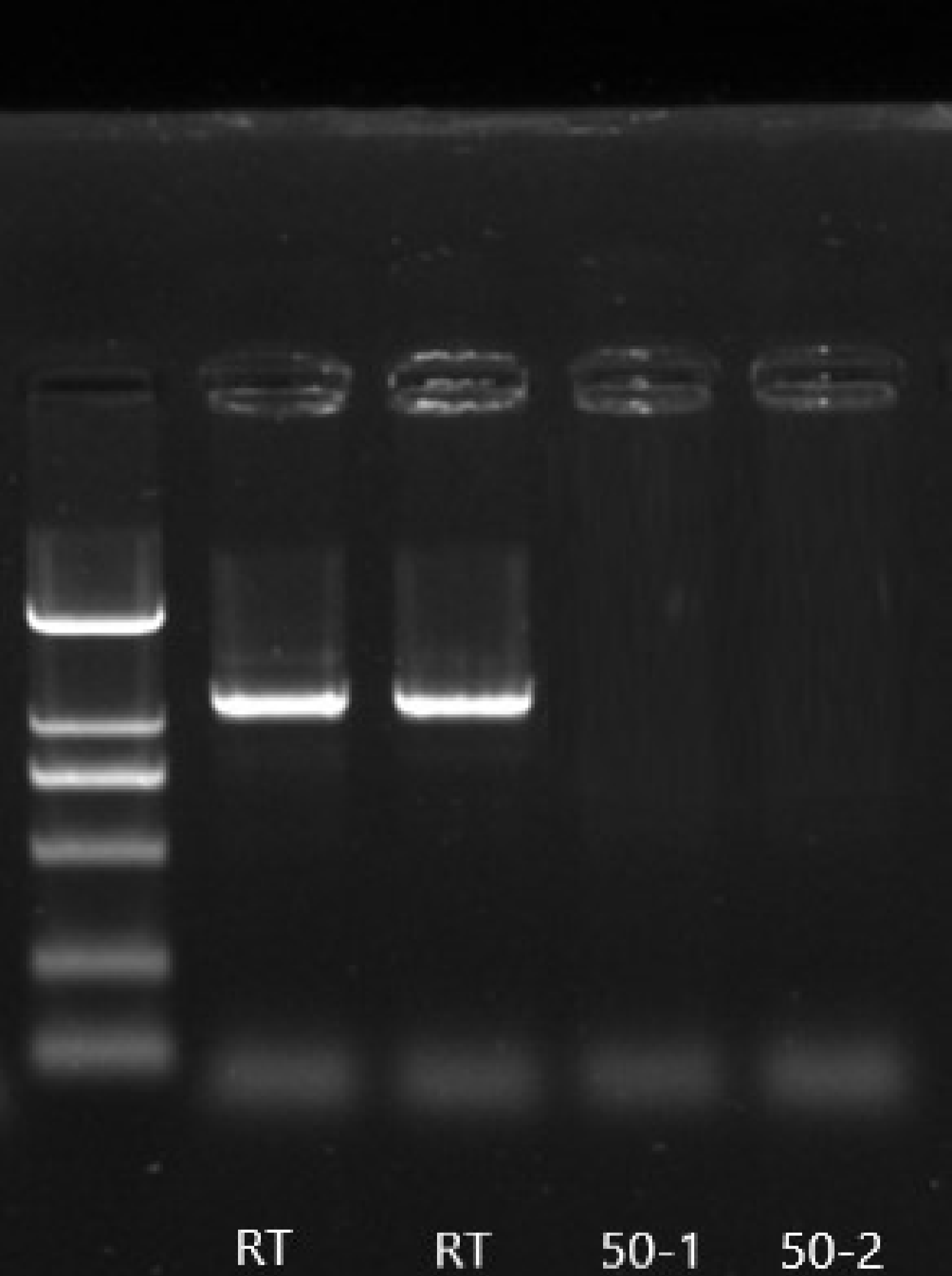

Fig. S5

Supplement: S1 Raw images — (PDF) [file pone.0281530.s014.pdf]
