# Supplementary material for: Identification and functional analysis of protein secreted by Alternaria solani
Source: PLoS One. 2023 Mar 6;18(3):e0281530. doi: 10.1371/journal.pone.0281530 (PMC9987770; doi:10.1371/journal.pone.0281530)
Supplement: S1 Table — (PDF) [file pone.0281530.s009.pdf]

S1 Table Bioinformatics-based identification of the AsCEP50 protein

Gene name	cDNA(bp)	amino acids	Singal peptide	Domain	Molecular weight(kDa)	pI	Instability index	Aliphatic index	GRAVY
AsCEP50	1134	377	1-57aa	Pro_Al_protease Trypsin	38.03	4.77	24.65	85.49	0.153
