# Supplementary material for: Identification and functional analysis of protein secreted by Alternaria solani
Source: PLoS One. 2023 Mar 6;18(3):e0281530. doi: 10.1371/journal.pone.0281530 (PMC9987770; doi:10.1371/journal.pone.0281530)
Supplement: S2 Table — The expression of Actin, SEN4, SAG12 and DHAR1 in N. benthamiana leaves injected with empty vector and Agrobacterium containing AsCEP50. (PDF) [file pone.0281530.s010.pdf]

S2 Table Expression levels of senescence- and oxidative stress-associated genes

		CT				$CT			2^(-$CT)			2^(-$CT)			2^(-$$CT)			2^(-$$CT)		
		ACTIN	SEN4	SAG12	DHAR1	SEN4	SAG12	DHAR1	SEN4	SAG12	DHAR1	SEN4	SAG12	DHAR1	SEN4	SAG12	DHAR1	SEN4	SAG12	DHAR1
EV	EV1	22.09	22.96	22.94	26.95	0.87	0.85	4.86	0.55	0.55	0.03	0.53	1.06	0.07	1.03	0.52	0.53	1.00	1.00	1.00
		21.54	22.89	23.01	26.89	1.35	1.47	5.34	0.39	0.36	0.02				0.74	0.34	0.38			
	EV2	23.71	24.45	23.11	27.18	0.74	-0.60	3.47	0.60	1.52	0.09				1.13	1.43	1.38			
		23.81	24.58	22.95	26.98	0.78	-0.85	3.17	0.58	1.81	0.11				1.10	1.71	1.71			
AsCEP50	50-1	25.67	24.17	22.59	24.64	-1.50	-3.08	-1.03	2.83	8.43	2.04	1.69	5.08	1.06	5.34	7.96	31.30	3.19	4.80	16.33
		25.47	24.57	22.87	24.59	-0.89	-2.60	-0.88	1.86	6.05	1.84				3.51	5.71	28.33			
	50-2	24.91	24.50	23.10	27.16	-0.41	-1.81	2.25	1.33	3.51	0.21				2.51	3.31	3.22			
		24.54	24.98	23.32	27.18	0.44	-1.22	2.63	0.74	2.33	0.16				1.39	2.20	2.47			
EV	EV1	21.91	22.81	22.98	27.13	0.90	1.07	5.22	0.54	0.48	0.03	0.57	1.38	0.05	0.94	0.34	0.52	1.00	1.00	1.00
		21.51	22.93	22.86	27.22	1.42	1.35	5.71	0.37	0.39	0.02				0.65	0.28	0.37			
	EV2	23.67	23.68	22.48	27.24	0.00	-1.19	3.57	1.00	2.28	0.08				1.74	1.65	1.62			
		23.43	24.82	22.17	27.11	1.39	-1.25	3.68	0.38	2.38	0.08				0.67	1.72	1.50			
AsCEP50	50-1	25.19	24.21	22.44	24.35	-0.98	-2.75	-0.84	1.97	6.71	1.79	1.72	4.83	0.98	3.44	4.85	34.37	3.01	3.49	18.74
		25.39	24.42	22.91	24.53	-0.97	-2.48	-0.86	1.96	5.57	1.82				3.42	4.02	34.88			
	50-2	24.51	24.43	22.32	27.43	-0.08	-2.19	2.92	1.06	4.55	0.13				1.84	3.29	2.54			
		24.67	23.74	23.36	27.27	-0.93	-1.31	2.61	1.91	2.48	0.16				3.33	1.79	3.15			
EV	EV1	20.85	22.62	22.69	26.94	1.77	1.84	6.09	0.29	0.28	0.01	0.21	0.43	0.02	1.41	0.64	0.88	1.00	1.00	1.00
		19.61	22.74	22.67	26.99	3.13	3.06	7.38	0.11	0.12	0.01				0.55	0.28	0.36			
	EV2	21.78	23.38	22.27	27.05	1.60	0.49	5.27	0.33	0.71	0.03				1.59	1.64	1.55			
		21.20	24.62	21.88	26.82	3.42	0.68	5.61	0.09	0.62	0.02				0.45	1.44	1.22			
AsCEP50	50-1	23.07	24.00	22.25	24.05	0.93	-0.82	0.99	0.52	1.76	0.50	0.67	1.95	0.37	2.53	4.06	30.05	3.23	4.49	22.22
		23.83	24.13	22.68	24.34	0.30	-1.15	0.51	0.81	2.21	0.70				3.90	5.10	41.85			
	50-2	23.67	24.24	22.13	26.22	0.57	-1.54	2.55	0.67	2.91	0.17				3.25	6.70	10.20			
		22.94	23.50	23.06	26.08	0.56	0.12	3.14	0.68	0.92	0.11				3.26	2.12	6.77			
