# Supplementary material for: Identification and functional analysis of protein secreted by Alternaria solani
Source: PLoS One. 2023 Mar 6;18(3):e0281530. doi: 10.1371/journal.pone.0281530 (PMC9987770; doi:10.1371/journal.pone.0281530)
Supplement: S3 Table — Lesion length and width of N. benthamiana leaves injected with empty vector and Agrobacterium containing AsCEP50. (PDF) [file pone.0281530.s011.pdf]

S3 Table Quantitative Data for Senescence

	Independent 1					Independent 2					Independent 3				
		EV	AsCEP50	EV	AsCEP50		EV	AsCEP50	EV	AsCEP50		EV	AsCEP50	EV	AsCEP50
Lesion Width	Repeat 1	0	2.83	0	3.78	Repeat 1	0	2.34	0	3.505	Repeat 1	0	2.43	0	3.39
Lesion Length		0	4.73				0	4.67				0	4.35		
Lesion Width	Repeat 2	0	2.3	0	3.35	Repeat 2	0	2.12	0	3.34	Repeat 2	0	2.26	0	3.29
Lesion Length		0	4.4				0	4.56				0	4.32		
Lesion Width	Repeat 3	0	2.03	0	2.825	Repeat 3	0	2.32	0	2.885	Repeat 3	0	2.27	0	3.015
Lesion Length		0	3.62				0	3.45				0	3.76		
Lesion Width	Repeat 4	0	2.3	0	3.26	Repeat 4	0	2.14	0	3.45	Repeat 4	0	2.38	0	3.43
Lesion Length		0	4.22				0	4.76				0	4.48		
Lesion Width	Repeat 5	0	2.82	0	2.865	Repeat 5	0	2.68	0	3.215	Repeat 5	0	2.31	0	3.22
Lesion Length		0	2.91				0	3.75				0	4.13		
Lesion Width	Repeat 6	0	2.93	0	3.075	Repeat 6	0	2.43	0	3.19	Repeat 6	0	2.27	0	3.4
Lesion Length		0	3.22				0	3.95				0	4.53		
Lesion Width	Repeat 7	0	2.36	0	3.24	Repeat 7	0	2.62	0	3.7	Repeat 7	0	2.59	0	3.485
Lesion Length		0	4.12				0	4.78				0	4.38		
Lesion Width	Repeat 8	0	2.04	0	2.58	Repeat 8	0	2.53	0	3.5	Repeat 8	0	2.29	0	3.52
Lesion Length		0	3.12				0	4.47				0	4.75		
