# Supplementary material for: Identification and functional analysis of protein secreted by Alternaria solani
Source: PLoS One. 2023 Mar 6;18(3):e0281530. doi: 10.1371/journal.pone.0281530 (PMC9987770; doi:10.1371/journal.pone.0281530)
Supplement: S4 Table — (PDF) [file pone.0281530.s012.pdf]

S4 Table Primer Amplification Efficiency

concentration		150ng/uL	mean	30ng/uL	mean	6ng/uL	mean	1.2ng/uL	mean	240pg/uL	mean	48pg/uL	mean	9.6pg/uL	mean
Actin	Myc1	22.45	22.60	24.96	25.10	29.58	29.54	32.02	31.82	34.69	35.28	36.68	36.76	37.35	37.42
	Myc2	22.82		25.27		29.58		31.58		36.07		36.85		37.38	
	Myc3	22.53		25.07		29.45		31.87		35.09		36.75		37.52	
AsCEP50	Myc1	26.00	25.96	28.04	28.08	30.63	30.28	33.85	32.05	31.53	31.56	32.31	32.38	30.66	31.43
	Myc2	26.00		28.08		29.95		30.33		31.60		32.42		31.97	
	Myc3	25.89		28.13		30.25		31.98		31.55		32.40		31.67	
concentration		9.6pg/uL	mean	48pg/uL	mean	240pg/u	mean	1.2ng/uL	mean	6ng/uL	mean	30ng/uL	mean	150ng/uL	mean
PP2A	Myc1	32.22	32.42	29.90	30.35	28.23	28.33	26.03	26.01	23.52	23.54	20.50	20.53	19.66	19.71
	Myc2	32.61		30.80		28.43		25.98		23.56		20.57		19.76	
SEN4	Myc1	38.61	38.41	35.09	35.31	32.33	32.17	30.07	30.15	27.41	27.42	25.26	25.19	23.57	23.54
	Myc2	38.21		35.52		32.02		30.24		27.43		25.11		23.51	
SAG12	Myc1	36.94	35.31	36.18	36.64	33.26	33.46	30.93	31.16	28.65	28.77	26.92	26.48	25.18	25.25
	Myc2	33.69		37.11		33.65		31.39		28.90		26.03		25.31	
DHAR1	Myc1	29.80	29.93	34.91	35.09	32.60	32.58	30.09	30.23	28.03	28.06	25.36	25.31		
	Myc2	30.06		35.27		32.56		30.36		28.10		25.25			
