# Supplementary material for: Identification and functional analysis of protein secreted by Alternaria solani
Source: PLoS One. 2023 Mar 6;18(3):e0281530. doi: 10.1371/journal.pone.0281530 (PMC9987770; doi:10.1371/journal.pone.0281530)
Supplement: S5 Table — (PDF) [file pone.0281530.s013.pdf]

S5 Table Primers used for this study

Primer	Sequence （5'→3'）	Purpose
AsCEP50 FL Primer-F	TTGTAAAACGACGGCCAG TGATGGAGCTCACCGGAT	Primers used to amplify the *AsCEP50* (FL/NSP) gene for ligation of pUC19
AsCEP50 FL Primer-R	CCTGCAGGTCGACTCTAG AGACCGCGGGTCAAGGTG	Primers used to amplify the AsCEP50 (FL/NSP) gene for ligation of pUC19
AsCEP50 NSP Primer-F	TTGTAAAACGACGGCCAG TGATGGCCCCTACCACGG	Primers used to amplify the AsCEP50 (FL/NSP) gene for ligation of pUC19
AsCEP50 FL Primer-F	ggactggtacccgggATGGAGCTC ACCGGATTCCT	Primers used to amplify the *AsCEP50* (FL/NSP) gene for ligation of pCAMBIA-1301
AsCEP50 FL Primer-R	cccttgctcaccatgACCGCGGGT CAAGGTGAGT	Primers used to amplify the AsCEP50 (FL/NSP) gene for ligation of pCAMBIA-1301
AsCEP50 NSP Primer-F	ggactggtacccgggATGGCCCCT ACCACGGCCGCC	Primers used to amplify the AsCEP50 (FL/NSP) gene for ligation of pCAMBIA-1301
AsCEP50 -LR Primer-F	aaacgacggccagtgACTCGTGTA TAGCGTCATCC	Primers for amplifying upstream sequences of *AsCEP50*
AsCEP50 -LR Primer-R	cccaaaaatgctcctAGGCGAGAT CCTAAGAAGAT	Primers for amplifying upstream sequences of *AsCEP50*
AsCEP50 -RR Primer-F	attccgtcaccagccGACTGTTGA GAGGGATATGG	Primers for amplifying downstream sequences of *AsCEP50*
AsCEP50 -RR Primer-R	aggtcgactctagagTCTATTCTAT TGCCGTGGTT	Primers for amplifying downstream sequences of *AsCEP50*
Hyg Primer-F	cttaggatctcgcctAGGAGCATTT TTGGGCTTGG	Primers for amplifying the *Hyg* gene
Hyg Primer-R	tccctctcaacagtcGGCTGGTGAC GGAATTTTCA	Primers for amplifying the *Hyg* gene
KN50 Primer-F	cttaggatctcgcctATGATTGAAC AAGATGGATT	Primers required for the construction of the AsCEP50 revertant strains
KN50 Primer-R	tccctctcaacagtcTCAGAAGAA CTCGTCAAGAA	Primers required for the construction of the AsCEP50 revertant strains
Signal peptide Primer-F	TTTTAATTAAGAATT	Primers used to amplify *AsCEP50* for ligation of pSUC2
Signal peptide Primer-R	TAGGGAGAACCTCGA	Primers used to amplify *AsCEP50* for ligation of pSUC2
qRT-PCR Primer-F	AGTCTTCCCTTCCATCGTC G	Fluorescent quantitative ACTIN primers
qRT-PCR Primer-R	CTTCTCCATGTCGTCCCAG T	Fluorescent quantitative ACTIN primers
qRT-PCR Primer-F	TGGCGGTTCTTTCTACTCT GG	Fluorescent quantitative *AsCEP50* primers
qRT-PCR Primer-R	GTTGACGGGCTGGAAGTA GG	Fluorescent quantitative *AsCEP50* primers
Primer-F	AACATGGGGTGAAGGCAG AG	Primers for amplifying the *SEN4* gene
Primer-R	ATTGCCAGGGACAAGCTT GA	Primers for amplifying the *SEN4* gene
Primer-F	GAGGACTCTTGGACACTG CC	Primers for amplifying the *SAG12* gene
Primer-R	GCAGACACCATCTTCTCCC C	Primers for amplifying the *SAG12* gene
Primer-F	CAACGGGCTCTTCTCACAC T	Primers for amplifying the *DHAR1* gene
Primer-R	GTCACCCACTTGTCGTCGA T	Primers for amplifying the *DHAR1* gene
